# Exogenous H_2_S Induces Hrd1 S-sulfhydration and Prevents CD36 Translocation via VAMP3 Ubiquitylation in Diabetic Hearts

**DOI:** 10.14336/AD.2019.0530

**Published:** 2020-03-09

**Authors:** Miao Yu, Haining Du, Bingzhu Wang, Jian Chen, Fangping Lu, Shuo Peng, Yu Sun, Ning Liu, Xiaojiao Sun, Dong Shiyun, Yajun Zhao, Yan Wang, Dechao Zhao, Fanghao Lu, Weihua Zhang

**Affiliations:** ^1^Department of Pathophysiology, Harbin Medical University, Harbin, China.; ^2^Department of Urologic Surgery, First Affiliated Hospital of Harbin Medical University, Harbin, China.; ^3^Department of Cardiology, First Affiliated Hospital of Harbin Medical University, Harbin, China.; ^4^Key Laboratory of Cardiovascular Medicine Research (Harbin Medical University), Ministry of Education, Harbin, 150086, China.

**Keywords:** hydrogen sulfide, Hrd1, VAMP3, diabetic cardiomyopathy, ubiquitylation, S-sulfhydration

## Abstract

Hydrogen sulfide (H_2_S) plays physiological roles in vascular tone regulation, cytoprotection, and ATP synthesis. HMG-CoA reductase degradation protein (Hrd1), an E3 ubiquitin ligase, is involved in protein trafficking. H_2_S may play a role in controlling fatty acid uptake in diabetic cardiomyopathy (DCM) in a manner correlated with modulation of Hrd1 S-sulfhydration; however, this role remains to be elucidated. The aim of the present study was to examine whether H_2_S can attenuate lipid accumulation and to explain the possible mechanisms involved in the regulation of the H_2_S-Hrd1/VAMP3 pathway. Db/db mice and neonatal rat cardiomyocytes treated with high glucose, palmitate and oleate were used as animal and cellular models of type 2 diabetes, respectively. The expression of cystathionine-γ-lyase (CSE), Hrd1, CD36 and VAMP3 was detected by Western blot analysis. In addition, Hrd1 was mutated at Cys115, and Hrd1 S-sulfhydration was examined using an S-sulfhydration assay. VAMP3 ubiquitylation was investigated by immunoprecipitation. Lipid droplet formation was tested by TEM, BODIPY 493/503 staining and oil red O staining. The expression of CSE and Hrd1 was decreased in db/db mice compared to control mice, whereas CD36 and VAMP3 expression was increased. NaHS administration reduced droplet formation, and exogenous H_2_S restored Hrd1 expression, modified S-sulfhydration, and decreased VAMP3 expression in the plasma membrane. Using LC-MS/MS analysis, we identified 85 proteins with decreased ubiquitylation, including 3 vesicle-associated membrane proteins, in the cardiac tissues of model db/db mice compared with NaHS-treated db/db mice. Overexpression of Hrd1 mutated at Cys115 diminished VAMP3 ubiquitylation, whereas it increased CD36 and VAMP3 expression and droplet formation. siRNA-mediated Hrd1 deletion increased the expression of CD36 in the cell membrane. These findings suggested that H_2_S regulates VAMP3 ubiquitylation via Hrd1 S-sulfhydration at Cys115 to prevent CD36 translocation in diabetes.

Type 2 diabetes is a strong risk factor for the development of cardiovascular disease and atherosclerosis, which is related to dyslipidemia. Recent evidence suggests that lipid accumulation within the myocardium may be involved in the progression of heart failure [1]. In healthy hearts, approximately 70-90% of fatty acids taken up are quickly oxidized, while only a small proportion are esterified into triacylglycerol (TAG) [2]. Some studies have verified that in an insulin-resistant state, TAG accumulates in the heart [3, 4]. This cardiac accumulation of TAG could be due to increased myocardial long-chain fatty acid (LCFA) uptake. Luiken et al. have shown that 50% of LCFA uptake is mediated by the LCFA transport protein fatty acid translocase FAT/CD36 [5]. Although some studies have revealed TAG accumulation to be associated with cardiac contractile dysfunction [6], the exact mechanisms responsible for the regulation of TAG accumulation remain unclear.

Additional evidence has demonstrated that intracellular membrane trafficking and exocytosis are controlled by several superfamily proteins, including N-ethylmaleimide sensitive factor (NSF); its cofactor, soluble NSF attachment protein (αSNAP); and SNAP receptors (SNAREs) [7]. SNAREs can be classified as vesicle-associated SNAREs (v-SNAREs) or target membrane-associated SNAREs (t-SNAREs) [8]. T-SNAREs and vesicle-associated membrane proteins (VAMPs) represent all members of the SNARE superfamily [9]. Seven VAMP isoforms have been identified; VAMP3 and VAMP4 are predominantly expressed in the heart and skeletal muscle. Notably, many studies have established the importance of ubiquitylation in controlling receptor endocytosis and endosomal sorting [10, 11].

Ubiquitylation is thought to be an important signal regulating intracellular trafficking [12]. The endoplasmic reticulum (ER) transmembrane E3 ubiquitin ligase HMG-CoA reductase degradation protein (Hrd1) has been shown to mediate the ubiquitylation of substrate proteins in yeast and mammalian cell lines [13].

Hydrogen sulfide (H_2_S), the third identified gasotransmitter after nitric oxide and carbon monoxide, is involved in a wide range of physiological and pathological processes. Our previous study suggested that H_2_S inhibits the ubiquitylation of superoxide dismutase (SOD) to promote the initiation of autophagy in diabetic cardiomyopathy [14]. Ji *et al.* demonstrated that H_2_S regulates Krüppel-like factor 5 (KLF5) transcriptional activity via specific protein S-sulfhydration to prevent myocardial hypertrophy [15]. To date, no information exists on the potential role of H_2_S in modifying Hrd1 or on the role of H_2_S in modulating LCFA uptake in diabetic cardiomyopathy. Our findings raise the possibility that H_2_S participates in VAMP3 degradation through Hrd1 S-sulfhydration in the cardiac tissues of db/db mice.

## MATERIALS AND METHODS

### Animal model and treatment method

Homozygous male and female db/db mice (Animal Laboratory Centre of Nanjing University) (n=50, 8 weeks old) and age-matched lean littermates (C57BL/6) (n=25) were used in this study. The mice were bred in a conventional animal facility with a 12-hour light/dark cycle. The mice were divided into four groups. Half of the db/db mice and their lean age-matched C57BL/6 littermates were treated with NaHS (80 μmol/kg, every two days) by intraperitoneal injection for 6, 12 or 20 weeks (6-, 12- or 20-week-treated mice, respectively). Simultaneously, the remaining half were injected with equal amounts of saline. The animal experiments were carried out following the recommendations of the Guide for the Care and Use of Laboratory Animals published by the China National Institutes of Health. The feeding regimen was approved by the Animal Care Committees of Harbin Medical University, China.

### Tissue collection

Before tissue collection, the mice were fasted overnight. Blood was collected from the orbits of the mice. The tissues were rapidly removed and frozen in liquid nitrogen. Cardiac sections were embedded into Tissue-Tek OCT compound (Sakura) for histology. The ventricles were rapidly dissected and processed as follows: (1) a small (3×1×1 mm) section from the ventricle was placed into fixation buffer for TEM analysis, (2) a 20-30 mg section was rapidly frozen for TAG analysis, (3) a 20-30 mg section was used to make frozen sections, and (4) the remainder was used for Western blot analysis.

### H_2_S level detection with 7-azido-4-methylcoumarin

The fluorescence response of H_2_S in cardiac tissues and neonatal rat cardiomyocytes (NRCMs) was tested with 7-azido-4-methylcoumarin (C-7Az, Sigma-Aldrich). This probe has been testified to selectively respond to H_2_S [16]. The samples were incubated with C-7Az (50 μmol/L) in phosphate-buffered saline (PBS) at 37 °C for 30 min and then washed three times with PBS. The fluorescence responses in the samples were observed with a fluorescence microscope (Olympus, XSZ-D2, Japan).

### BODIPY 493/503 staining

Lipid droplets were stained with BODIPY 493/503 (Life Technologies) diluted in 10% (v/v) dimethyl sulfoxide in PBS to a concentration of 1 mg/mL [17]. Frozen cardiac tissue or NRCMs were fixed with 4% paraformaldehyde for 20 min and then immersed in BODIPY 493/503 solution for 30 min at 37 °C. After the samples were washed 3 times with PBS, the stained droplets were observed using a fluorescence microscope (Olympus, XSZ-D2, Japan).

### Oil red O staining

After induction of lipid droplets, cells were stained with oil red O using an adipogenesis assay kit from the Nanjing Jiancheng Bioengineering Institute (Nanjing, China). The cells were fixed with 4% paraformaldehyde for 10 min and then incubated with oil red O solution for 20 min at room temperature. The stained lipids were observed using a fluorescence microscope (Olympus, XSZ-D2, Japan).

### Neonatal rat cardiomyocyte culture

Cultures of NRCMs were prepared according to previously described methods [18]. Hearts were collected from 2- to 3-day-old neonatal Sprague-Dawley rats (Animal Research Institute of Harbin Medical University, China) and washed in D-Hanks balanced salt solution (mmol/L: 0.4 KCl, 0.06 KH_2_PO_4_, 8.0 NaCl, 0.35 NaHCO_3_, and 0.06 Na_2_HPO_4_·7H_2_O, pH 7.2) at 4 °C. Then, the tissue was cut into pieces and incubated with 0.25% trypsin at 37 °C for 8 min. An equal volume of cold Dulbecco’s modified Eagle’s medium (DMEM) containing 10% (v/v) fetal bovine serum was added to terminate the digestion. The digestion step was repeated six times. The supernatant cells were filtered and isolated by centrifugation (2000 × *g*, 4 °C, 10 min). The cells were resuspended in DMEM containing 10% (v/v) fetal bovine serum, 100 U/mL penicillin and 100 µg/mL streptomycin and incubated at 37 °C in humidified air containing 5% CO_2_. After 1 hour of culture at 37 °C, the unattached cells were transferred to fresh medium. The medium was replaced with fresh culture medium periodically.

### Cell experiment

NRCMs were divided into six groups: (1) a control group (low glucose, 5.5mM); (2) a high glucose (HG)+palmitate (Pal)+oleate (Ole)-treated group (HG, 40 mM; Pal, 200 μM; Ole, 200 μM); (3) an HG+Pal+Ole+NaHS (H_2_S donor, 100 μM)-treated group; (4) an HG+Pal+Ole+propargylglycine (PPG, a CSE inhibitor, 10 nM)-treated group; (5) an HG+Pal+Ole+dithiothreitol (DTT, a reducer of disulfide bonds, 1mM)-treated group; and (6) an HG+Pal+Ole+PYR41 (a ubiquitin activating enzyme inhibitor, 50μM)-treated group. The reagents were added to the culture medium for 24 or 48 hours.

### Western blot analysis

The cultured cells and cardiac tissues were solubilized in the presence of protease inhibitors as previously described [19]. After quantification using a BCA Protein Assay Kit (Solarbio), the proteins were separated by SDS-PAGE and transferred to nitrocellulose membranes (Pall Corporation, Pensacola, FL). The membranes were incubated with the appropriate primary antibodies; the primary antibodies were then detected using peroxidase-conjugated secondary antibodies (1:5000), which were visualized with an enhanced chemiluminescence reagent (ECL, GE Healthcare, Amersham, UK). Immunoblotting was performed with the following antibodies: anti-CSE (1:1000, Proteintech Group, USA), anti-CD36 (1:1000, Proteintech Group, USA), anti-VAMP3 (1:1000, Proteintech Group, USA), anti-Hrd1 (1:1000, Proteintech Group, USA), anti-β-tubulin (1:1000, Proteintech Group, USA), and anti-caveolin (1:1000, Proteintech Group, USA). Densitometry was conducted through image processing, and the data were analyzed with AlphaView SA.

### Immunostaining

Cells were fixed in 4% paraformaldehyde for 30 min and then permeabilized with 0.5% Triton X-100 for 30 min (the permeabilization step was omitted when staining for proteins on the cell membrane). The coverslips of cells were blocked with 5% BSA for 1 hour at 37 °C. The cells were then incubated with anti-CD36 antibodies (1:1000, Proteintech Group, USA) and anti-VAMP3 antibodies (1:1000, Proteintech Group, USA) at 4 °C overnight before being incubated for 2 hours with anti-rabbit IgG or anti-mouse IgG. Images were captured and analyzed with a Zeiss LSM 510 inverted confocal microscope.

### Cell plasma membrane protein isolation

Cell plasma membrane proteins were isolated from 1×10^7^ cells or 50 µg of cardiac tissue using a Nuclear-Cytosol-Mem Extraction Kit (Applygen) following the manufacturer’s instructions. The isolated protein fractions were analyzed by Western blotting.

### Immunoprecipitation

Protein samples were incubated with agarose (Protein G Plus Agarose, Santa) at 4 °C for 20 min and centrifuged at 10,000 × *g* for 3 min. Agarose and antibodies (anti-Hrd1 or anti-VAMP3) were added, and the samples were incubated with gentle rotation overnight. The precipitates were collected after centrifugation at 10,000 × *g* for 5 min and three washes with buffer. Samples meeting the purity standards were subjected to Western blotting with anti-VAMP3 or anti-ubiquitin antibodies.

### S-sulfhydration assay

Briefly, cells were sonicated in buffer containing 250 mM HEPES buffer (pH 7.7), 1 mM EDTA, 0.1 mM neocuproine and 100 μM deferoxamine and then centrifuged at 13,000 × *g* for 30 min at 4 °C. The cell lysates were incubated with blocking buffer (sonication buffer with 2.5% SDS and 20 mM MMTS) for 20 min at 50 °C with frequent vortexing. The proteins were precipitated with acetone at -20 °C for 20 min, and then the samples were resuspended in blocking buffer (with 1% SDS) following the addition of 4 mM biotin-HPDP. After incubation for 3 hours at 25 °C, the biotinylated proteins were mixed with streptavidin-agarose beads. Then, the cells were washed with HENS buffer. The biotinylated proteins were eluted by SDS-PAGE and subjected to Western blotting using an anti-Hrd1 antibody.

### Measurement of intracellular levels of polysulfide

Intracellular production of polysulfide was examined with a new type of fluorescent probe, SSP4 (Dojindo, Japan). Briefly, isolated cells were incubated with 50 μmol/L SSP4 in serum-free DMEM containing 0.003% Cremophor EL at 37 °C for 15 min in the dark. After washing, SSP4 was detected using a fluorescence microscope (Olympus, XSZ-D2).

### Point mutation

Adenoviruses expressing GFP and Hrd1 were purchased from Cyagen Biosciences Inc (Guangzhou, China). Full-length mouse Hrd1 with a single mutation of cysteine 115 to alanine and GFP cDNA were inserted into a pM vector (Cyagen Biosciences) between the Kozak and T2A sites. Subconfluent (70%) monolayers of cells were transfected with the pHrd1-cys-115-GFP plasmid in Opti-MEM reduced-serum medium (Gibco^®^, life technology) using Lipofectamine 3000 reagent (Invitrogen). The adenoviruses were added to NRCMs directly, and after 4 hours of transfection, the medium was replaced with fresh medium. The NRCMs were treated with different reagents after 24 hours, and the related proteins were detected by Western blotting.

### siRNA transfection

H9c2 cells (Organism Rattus norvegicus Disease Comments Breed/subspecies: BDIX. Proper Citation ATCC Cat# CRL-1446) (80% confluent) were treated with Hrd1 short interfering RNAs (siRNAs) (Sangon Biotech Shanghai) for 48 hours to inhibit Hrd1 expression according to the manufacturer’s instructions. Transfection was performed with Lipofectamine 3000 (Invitrogen). Briefly, transfection reagent and Hrd1 siRNAs were mixed and incubated for 20 min to form complexes, which were then added to plates containing cells and medium. For further analysis, the cells were incubated in a CO_2_ incubator at 37 °C.

### Statistical analysis

The results were analyzed with the Prism software package (GraphPad Software). The results are shown as the mean ± standard deviation (SD). Differences among three or more groups were analyzed using one-way ANOVA and Bonferroni’s correction. Differences between individual groups were analyzed using Student’s t-test.

## RESULTS

### Characteristics of db/db mice

The body and heart weights of the 12- and 20-week-treated db/db mice were significantly higher than those of their age-matched lean littermates. The blood glucose concentrations, plasma free fatty acid concentrations and fatty acid β-oxidation levels were also elevated in the db/db mice compared with the lean controls. In addition, measurement of plasma insulin concentrations and glucose tolerance revealed that the db/db mice were markedly hyperinsulinemic. These results demonstrated that db/db mice recapitulated the hallmark features of type 2 diabetes. Fatty acid uptake was further measured using fluorescently labeled fatty acids (BODIPY 558/568C_12_) and was significantly increase in the HG+Pal+Ole (with or without PPG)-treated groups compared with the NaHS-treated and control groups. We also examined beta-oxidation and glucose uptake levels, and our results showed that fatty acid beta-oxidation was increased and that glucose uptake was slightly decreased in NRCMs in response to HG, Pal and Ole treatment for 24 or 48 hours. These results indicated that under diabetic conditions, the heart is forced to increase the uptake of fatty acids instead of glucose. (Supplementary Fig. 1).

In addition, we detected the effects of H_2_S on cardiac functions. Our echocardiography results showed that the ejection fraction (EF) and fraction shortening (FS) were decreased at 12 weeks and 20 weeks in db/db mice compared to control mice, whereas they improved after treatment with NaHS. All the results indicated that cardiac pump function was reduced in db/db mice. (Supplementary Fig. 2).

### H_2_S production in db/db mice

**Figure 1.**
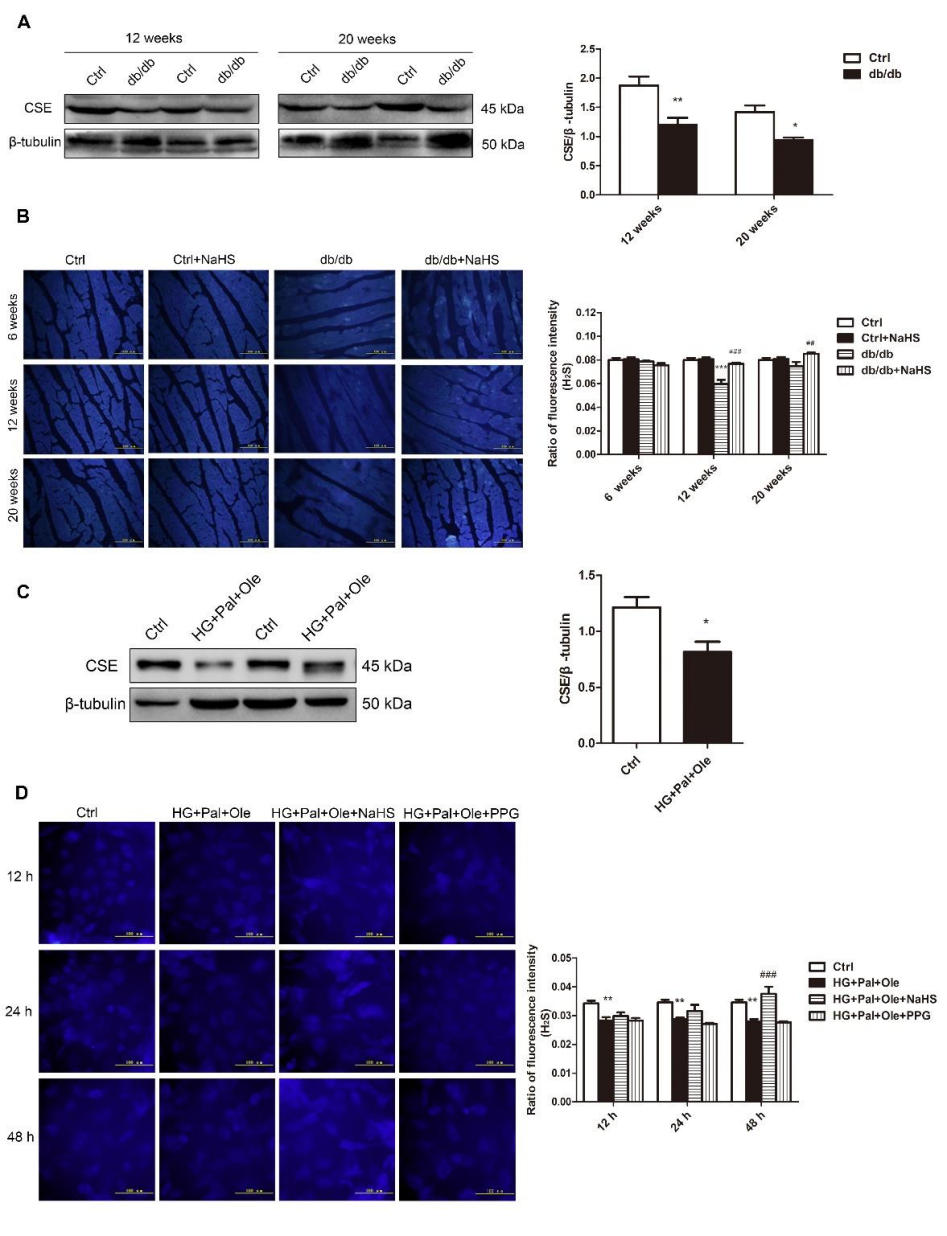
H_2_S levels in the hearts of db/db mice. (A) CSE expression in the cardiac tissues of db/db mice. (B) Fluorescence microscopy was used to detect H_2_S levels in cardiac tissues with the fluorescent probe 7-azido-4-methyl-coumarin (C-7Az). (C) CSE expression in neonatal rat cardiomyocytes (NRCMs) treated with high glucose, palmitate, and oleate. (D) H_2_S levels were examined at 12, 24, and 48 hours with the fluorescent probe C-7Az. Values are presented as the mean± SD from n=6 replicates. *p<0.05, **p<0.01, ***p<0.001 vs control group, ##*p*<0.01, ###*p*<0.001 vs db/db group or HG+Pal+Ole group.

Our previous study showed that endogenous H_2_S production was decreased in streptozotocin (STZ)-induced diabetic cardiomyopathy. In this study, we tested the expression of cystathionine-γ-lyase (CSE), a principal enzyme for H_2_S production, in the hearts of db/db mice (Fig. 1A). As expected, the expression of CSE was downregulated in db/db mice compared to control mice. We also detected H_2_S levels in mouse hearts using the H_2_S probe C-7Az. The results revealed that H_2_S levels were reduced in the hearts of the db/db mice but recovered after NaHS injection (Fig. 1B).

NRCMs were treated with HG, Pal and Ole to mimic the environment of type 2 diabetes, and the results confirmed that H_2_S levels and CSE expression were reduced in the HG+Pal+Ole group compared to the control group; however, these effects were abolished by treatment with NaHS (Fig. 1C, D).

### Intramyocellular lipid droplet formation

**Figure 2.**
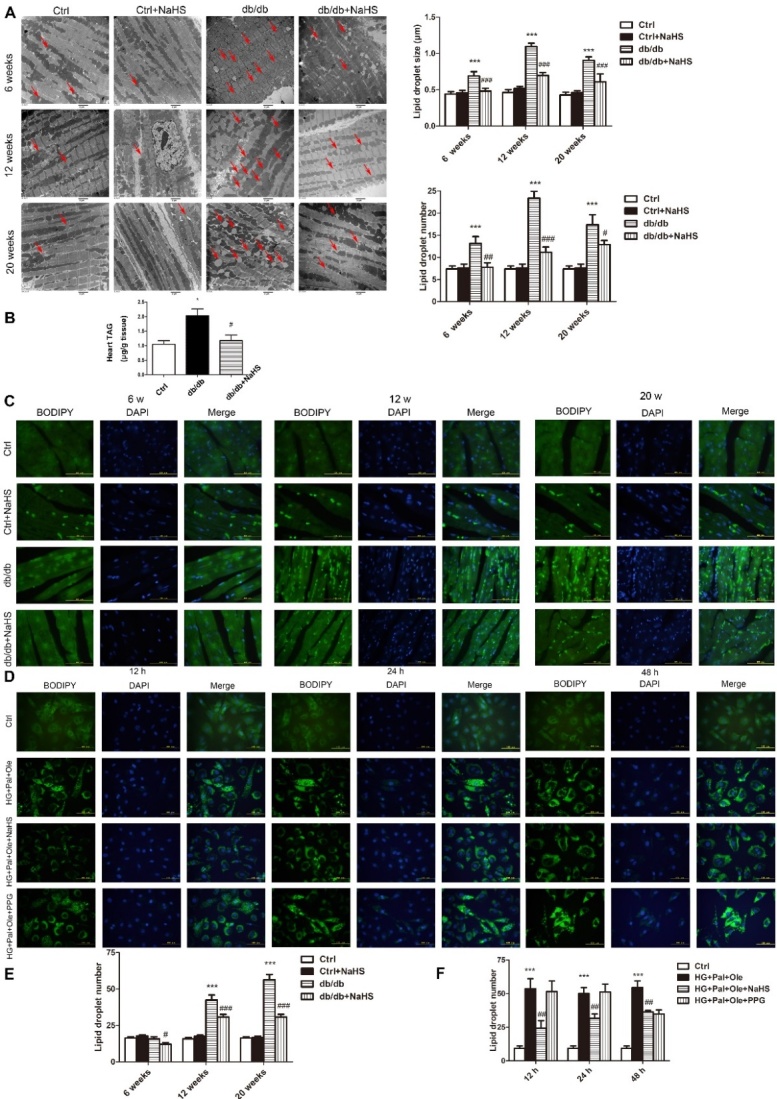
Exogenous H_2_S reduces the numbers and sizes of lipid droplets in the cardiac tissues of db/db mice. (A) Transmission electron microscopy (TEM) of cardiac tissues from db/db mice treated for 6, 12, and 20 weeks. Lipid droplets are indicated by the red arrows. (B) The content of triacylglycerol in cardiac tissue of 20-week-treated db/db, control and db/db+NaHS mice. (C) Lipid droplets in cardiac tissues of db/db mice detected with the fluorescent probe BODIPY 493/503. (D) Lipid droplets in NRCMs detected with the fluorescent probe BODIPY 493/503. (E) Statistics analysis of lipid droplets number in cardiac tissues of db/db mice. (F) Statistics analysis of lipid droplets number in NRCMs. Values are presented as the mean± SD from n=5 replicates. *p<0.05, **p<0.01, ***p<0.001 vs control group, #*p*<0.05, ##*p*<0,01, ###*p*<0.001 vs db/db group or HG+Pal+Ole group.

To examine droplet formation in the hearts of db/db mice treated with NaHS, TEM was used to detect the sizes and numbers of droplets at different times in cardiac tissues of these mice. Our results revealed that the lipid droplets were larger and more numerous in the model db/db group than in the NaHS-treated db/db group or the control group (Fig. 2A). The cardiac TAG levels of model db/db mice were also higher than those of NaHS-treated db/db mice and control mice (Fig. 2B). As shown in Fig. 2C, staining of neutral fats with BODIPY 493/503 also confirmed that there were more droplets in model db/db mice than in NaHS-treated db/db mice or control mice.

To further investigate whether exogenous H_2_S affects free fatty acid uptake, we examined droplet formation in cultured cardiomyocytes over time. We used the BODIPY 493/503 probe to detect the numbers and sizes of the droplets. Our results showed that at 12, 24 and 48 hours, there were more droplets in the HG+Pal+Ole group than in the HG+Pal+Ole+NaHS group or the control group (Fig. 2D). However, we also found that administration of PPG, an inhibitor of CSE, could increase the number of droplets. These results demonstrated that H_2_S could regulate droplet formation.

### Sarcolemmal and intracellular CD36 and VAMP3 protein content and localization

**Figure 3.**
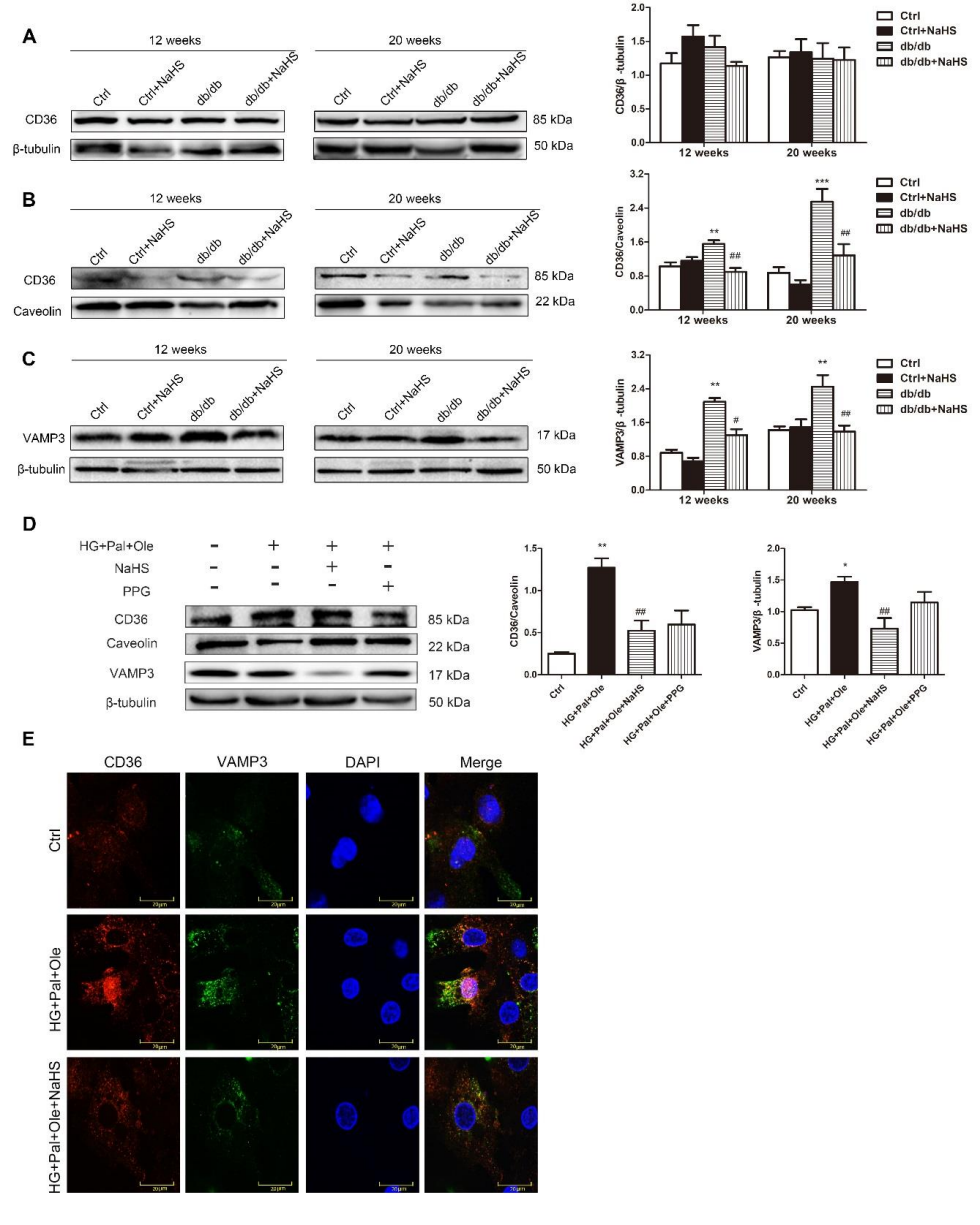
Exogenous H_2_S decreases CD36 translocation to the cell membrane and VAMP3 expression. Western blot analysis was used to detect CD36 expression in total cell lysates (A) and cell membranes (B) and to detect VAMP3 expression in the cytoplasm in cardiac tissues from db/db mice (C). (D) CD36 expression in the cell membrane and VAMP3 expression in the cytoplasm of NRCMs were also detected by Western blot analysis. The quantified levels of CD36 and VAMP3 are also presented. Values are presented as the mean± SD from n=5 replicates. *p<0.05, **p<0.01, ***p<0.001 vs control group, #*p*<0.05, ##*p*<0.01 vs db/db group or HG+Pal+Ole group. (E) The expression and colocalization of CD36 (red fluorescence) and VAMP3 (green fluorescence) were also investigated.

CD36, which is located in the cell membrane, is known to mediate LCFA uptake. Previous studies have identified proteins that mediate regulated exocytosis in cardiomyocytes, such as VAMP3 [20], VAMP7/8 [21], and syntaxin [22]. Most of them belong to a superfamily of transmembrane proteins named SNAREs, which mediate the trafficking of cellular materials between intracellular compartments [23]. The SNARE protein VAMP3 is known to be involved in vesicle fusion and trafficking [24]. The total protein content of CD36 in the hearts of the model db/db mice was not significantly different from that in the NaHS-treated db/db mice. However, the cell membrane CD36 protein content was elevated in myocytes from model db/db mice compared with myocytes from NaHS-treated db/db mice or control mice. Concomitantly, the intracellular VAMP3 protein levels were significantly higher in the model db/db mice than in the NaHS-treated db/db mice (Fig. 3A-C).

In NRCMs, we found that the membrane-bound CD36 and intracellular VAMP3 expression levels were higher in the HG+Pal+Ole group than in the HG+Pal+Ole+NaHS group (Fig. 3D). To further detect whether CD36 and VAMP3 were translocated to the cell membrane, we used immunofluorescence to detect CD36 (red) and VAMP3 (green) expression levels. The results showed that the expression and cell membrane translocation of CD36 and VAMP3 were increased in the HG+Pal+Ole group (Fig. 3E).

### The effect of exogenous H_2_S on VAMP3 ubiquitylation in db/db mice

**Figure 4.**
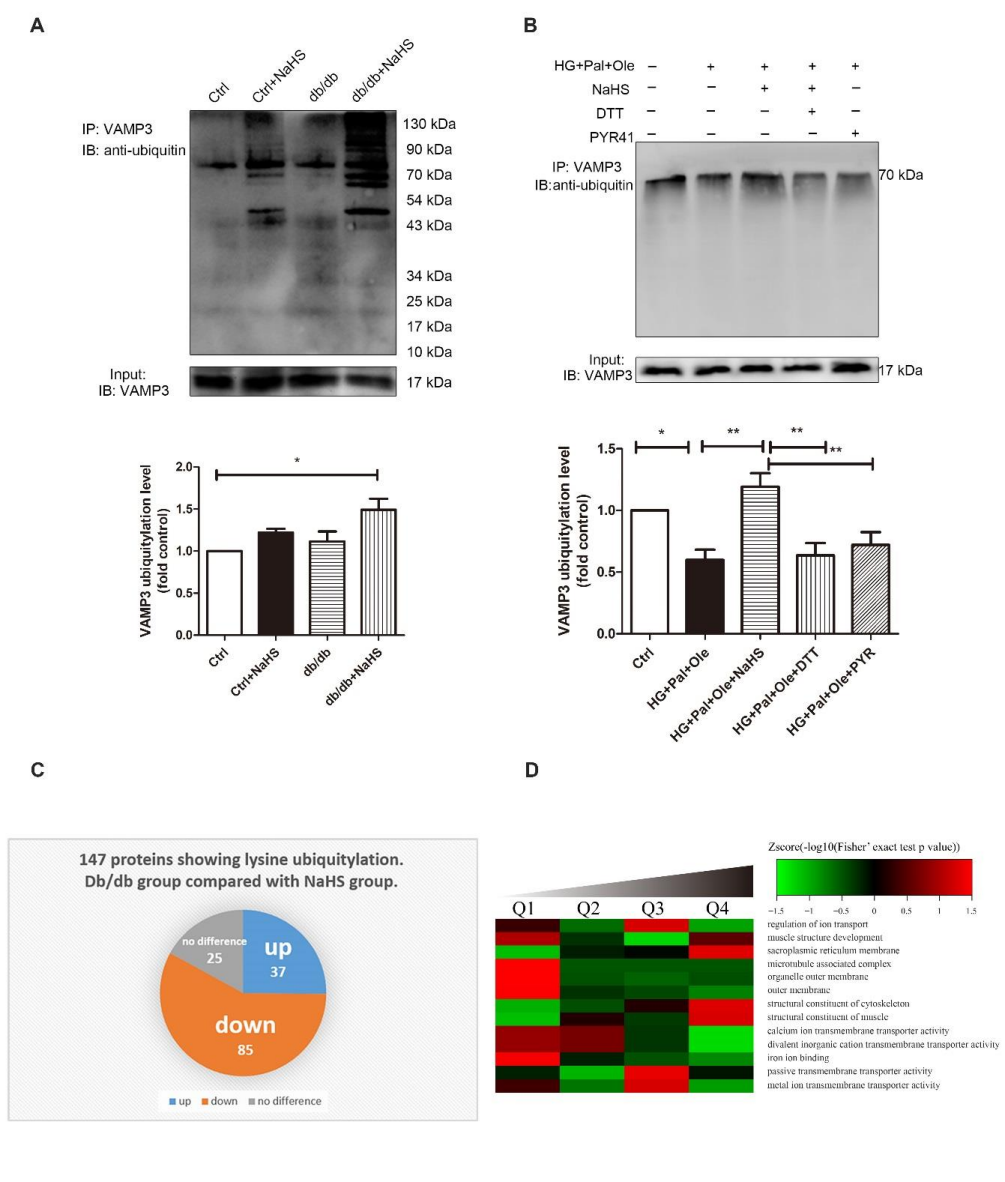
Exogenous H_2_S regulates ubiquitylation levels in db/db mouse cardiac tissues. (A) Cardiac tissue lysates were immunoprecipitated with an anti-VAMP3 antibody and immunoblotted with an antibody specific for ubiquitin. (B) Neonatal rat cardiomyocytes were treated with high glucose, palmitate, oleate, NaHS, DTT, and/or PYR41, and the ubiquitylation levels of VAMP3 were detected by immunoprecipitation. Values are presented as the mean± SD from n=3 replicates. *p<0.5, **p<0.01. (C) Pie chart illustrating the ubiquitylation statuses of proteins identified by LC-MS/MS following immunoprecipitation from cardiac tissues with lysine ubiquitylation (Kub) antibodies in model db/db mice compared to NaHS-treated db/db mice. (D) Molecular functions were identified by bioinformatic analysis. The quantified Kub proteins in this study were divided into four quantiles according to the quantification ratio: Q1 (0~15%), Q2 (15~50%), Q3 (50~85%) and Q4 (85~100%). The relative fold change was calculated for db/db model mice versus db/db+NaHS mice. The green color indicates a Zscore < 0, with a ratio less than the average. The red color indicates a Zscore > 0, with a ratio greater than the average.

VAMP3 was heavily ubiquitylated at Lys66, Lys68 and Lys77. Our evidence demonstrated that the ubiquitylation levels of VAMP3 in the hearts of the NaHS-treated db/db mice were higher than those in the model db/db mice and control mice (Fig. 4A). Similarly, the ubiquitylation of VAMP3 was upregulated in NRCMs treated with HG+Pal+Ole+NaHS. When DTT (1 mmol/L) and PYR41 (50 μmol/L) were added to reverse sulfhydration and to inhibit ubiquitin-activating enzyme E1, respectively, the ubiquitylation levels of VAMP3 were downregulated (Fig. 4B). To further reveal the function of exogenous H_2_S in modulating cardiac ubiquitylation in db/db mice, we used LC-MS/MS proteomic analysis to detect lysine ubiquitylation. More than 147 identified proteins showed lysine ubiquitylation, of which 85 proteins were hypoubiquitylated in the hearts of the model db/db mice compared with the hearts of NaHS-treated db/db mice (Fig. 4C). The results of the molecular function-based cluster analysis are shown in Fig. 4B. It was found that proteins involved in transporter activity, molecular transducer activity, structural molecular activity and protein binding factor activity were among those downregulated in the cardiac tissues of model db/db mice compared with NaHS-treated db/db mice (Fig. 4D).

### Hrd1 is required for VAMP3 degradation

**Figure 5.**
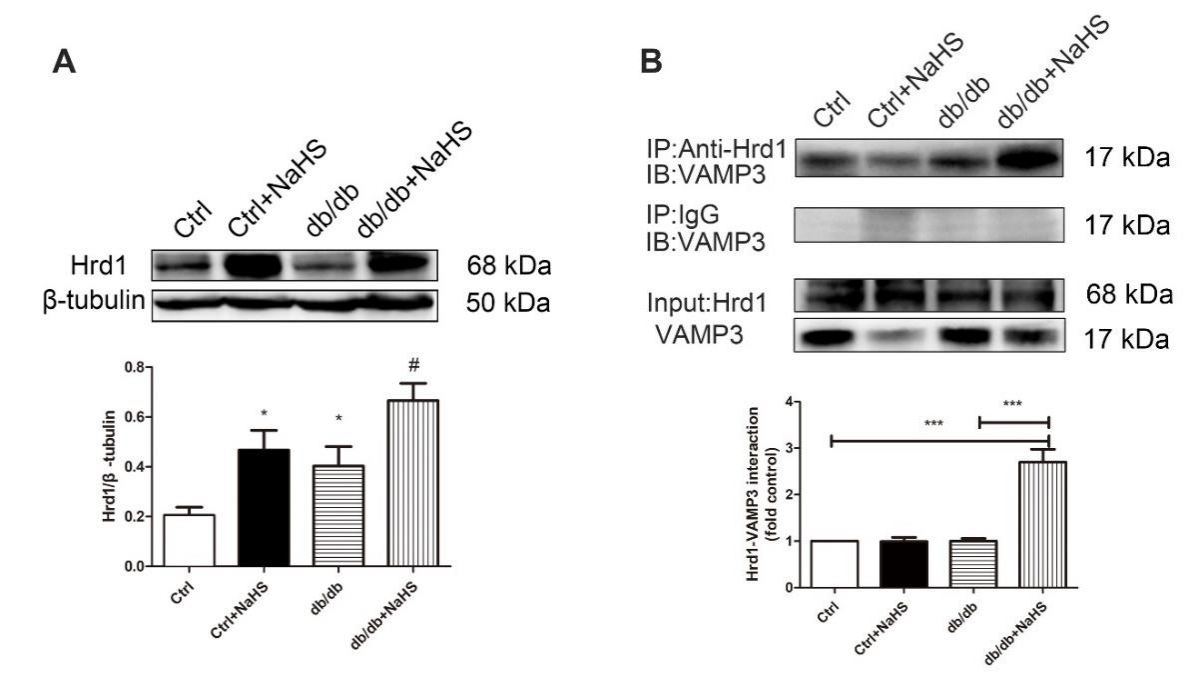
The effects of H_2_S on the Hrd1/VAMP3 pathway in db/db mouse hearts. (A) The expression of Hrd1 was detected by Western blot analysis in mouse myocardia. Values are presented as the mean± SD from n=5 replicates. *p<0.05 vs control group, #*p*<0.05 vs db/db group. (B) The interaction of Hrd1 with VAMP3 was detected by immunoprecipitation and Western blot analysis. Values are presented as the mean± SD from n=5 replicates. ***p<0.001.

Hrd1, as an E3 ubiquitin ligase, interacts with a specific substrate to target proteins for degradation. In this study, cardiac Hrd1 expression was downregulated in model db/db mice compared with NaHS-treated db/db mice (Fig. 5A). Considering the association of Hrd1 with VAMP3, we reasoned that Hrd1 might be required for VAMP3 ubiquitylation. Our results confirmed the interaction between Hrd1 and VAMP3 (Fig. 5B).

**Figure 6.**
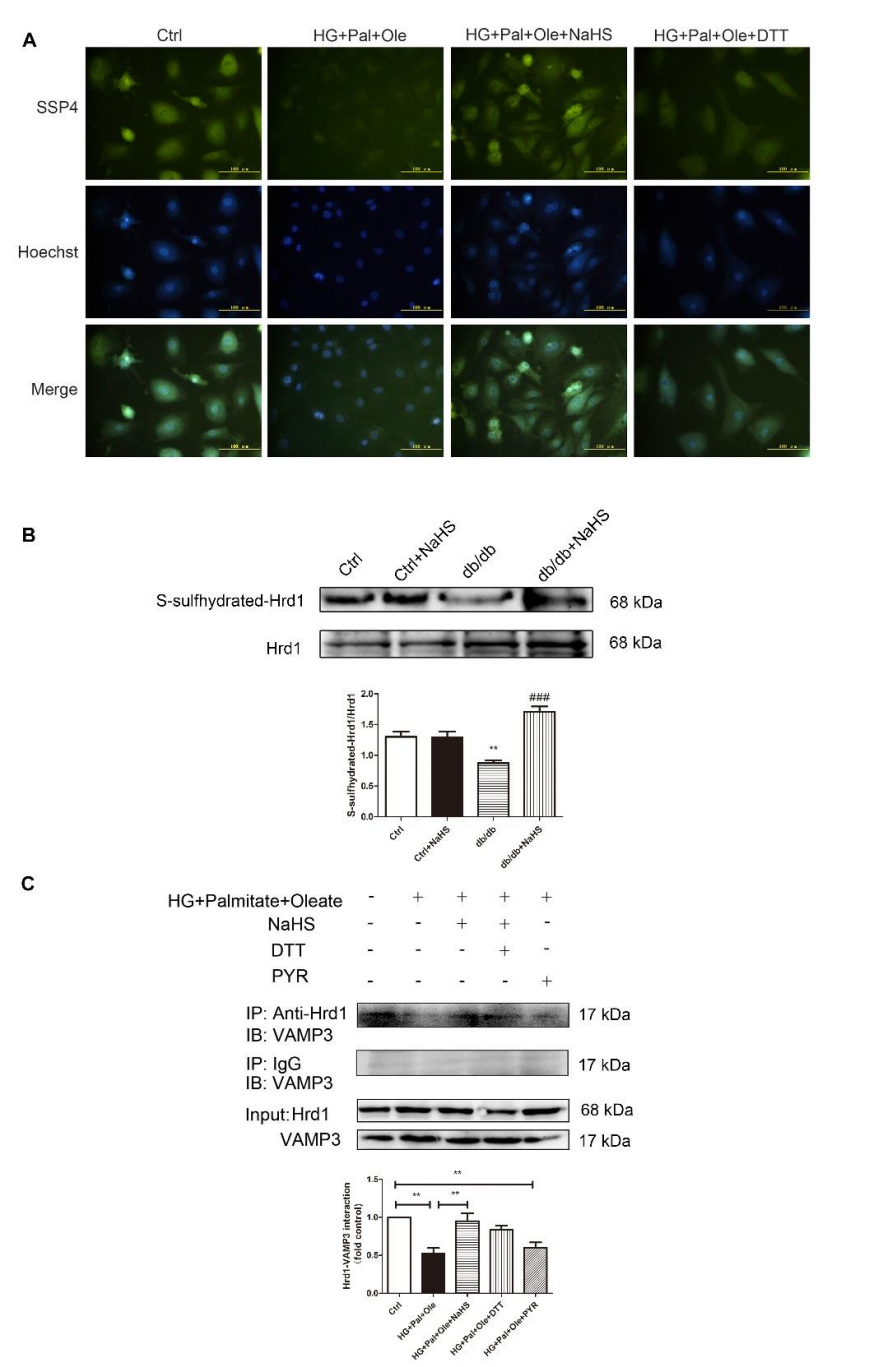
Exogenous H_2_S regulates Hrd1 S-sulfhydration in NRCMs. (A) The intracellular levels of polysulfide in NRCMs were detected with the fluorescent probe SSP4. (B) S-sulfhydration on Hrd1 was detected with the biotin switch (S-sulfhydration) method. Values are presented as the mean± SD from n=4 replicates. **p<0.01 vs control, ###*p*<0.001 vs db/db group. (C) The interaction of Hrd1 with VAMP3 was detected after treatment with DTT and PYR41 by immunoprecipitation and Western blot analysis. Values are presented as the mean± SD from n=3 replicates. **p<0.01.

It has been reported that S-sulfhydration, the addition of one sulfhydryl or persulfide group, is a novel posttranslational modification by H_2_S in eukaryotic cells. To confirm whether exogenous H_2_S affects the interaction between Hrd1 and VAMP3 via S-sulfhydration modification, we used the fluorescent probe SSP4 to detect intracellular protein polysulfide levels. DTT (1 mmol/L), a reducing agent that reverses sulfhydration, was used to treat cardiomyocytes for 10 min. The results showed that exogenous H_2_S enhanced SSP4 fluorescence intensity, suggesting that polysulfide could accumulate after H_2_S application (Fig. 6A).

To further investigate the effect of H_2_S on Hrd1 by S-sulfhydration, we detected the S-sulfhydration of the Hrd1 protein. The biotin switch assay revealed that exogenous H_2_S could regulate Hrd1 S-sulfhydration (Fig. 6B). Furthermore, treatment with DTT and PYR41 decreased the interaction between Hrd1 and VAMP3 (Fig. 6C).

### Exogenous H_2_S sulfhydrated Hrd1 at Cys115 to regulate VAMP3 ubiquitylation

**Figure 7.**
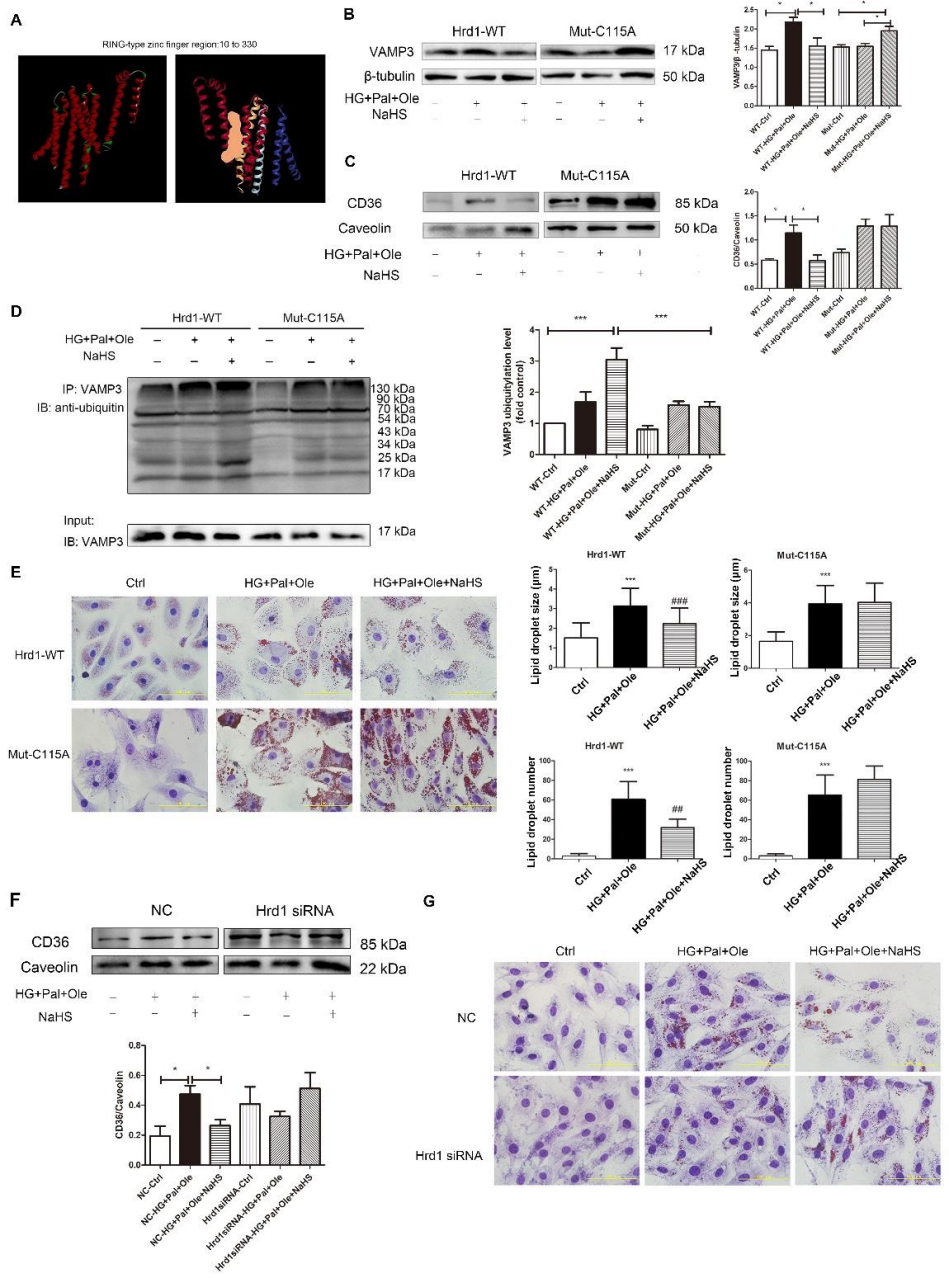
Exogenous H_2_S sulfhydration of Hrd1 at Cys115 prevents VAMP3 degradation. (A) 3D model of wild-type Hrd1 and its activity center (orange area: RING-type zinc finger region). After transfection with plasmids containing wild-type Hrd1 or Hrd1 mutated at Cys115 for 24 hours, NRCMs were pretreated with NaHS (100 µM) for 4 hours before treatment with high glucose, palmitate and oleate for 24 hours. The expression levels of VAMP3 (B) and CD36 (C) were measured, and the ubiquitylation level of VAMP3 (D) was also detected by immunoprecipitation. Data are presented as the mean± SD from n=6 replicates. *p<0.05, ***p<0.001 (E) The numbers and sizes of droplets in NRCMs were detected with oil red O. Data are presented as the mean± SD from n=6 replicates. ***p<0.001 vs control group, ##*p*<0,01, ###*p*<0.001 vs HG+Pal+Ole group. (F) Hrd1 was knocked down with siRNA in H9c2 cells. The expression of CD36 in the cell membrane was detected. Data are presented as the mean± SD from n=6 replicates. *p<0.05. (G) siRNA-mediated silencing of Hrd1 increased the numbers of lipid droplets in H9c2 cells treated with NaHS, as determined by oil red O staining.

To investigate how Hrd1 interacts with VAMP3 and to elucidate which specific cysteine residues may be responsible for this interaction, we conducted a computational modeling study of Hrd1. The optimized structure of Hrd1 is depicted in Fig. 7A. We used Discovery Studio software to detect the Hrd1 activity center (Fig. 7A). Next, we transfected NRCMs with wild-type Hrd1 or Hrd1 in which Cys115 was mutated to Ala (Supplementary Fig. 3). After transfection, exogenous H_2_S could not reduce the expression levels of VAMP3 or CD36 in the group overexpressing mutant Hrd1 and treated with Pal and Ole (Fig. 7B, C). We also found that the ubiquitylation level in the mutant Hrd1-overexpressing group treated with NaHS was clearly lower than that in wild-type Hrd1-overexpressing group treated with NaHS (Fig. 7D). Staining of neutral fats with oil red O also confirmed that the size and number of droplets were not reduced in the mutant Hrd1 group treated with NaHS (Fig. 7E). The above results demonstrated that exogenous H_2_S modified Hrd1 at the Cys115 site.

### Hrd1 siRNA increased the formation of lipid droplets

**Figure 8.**
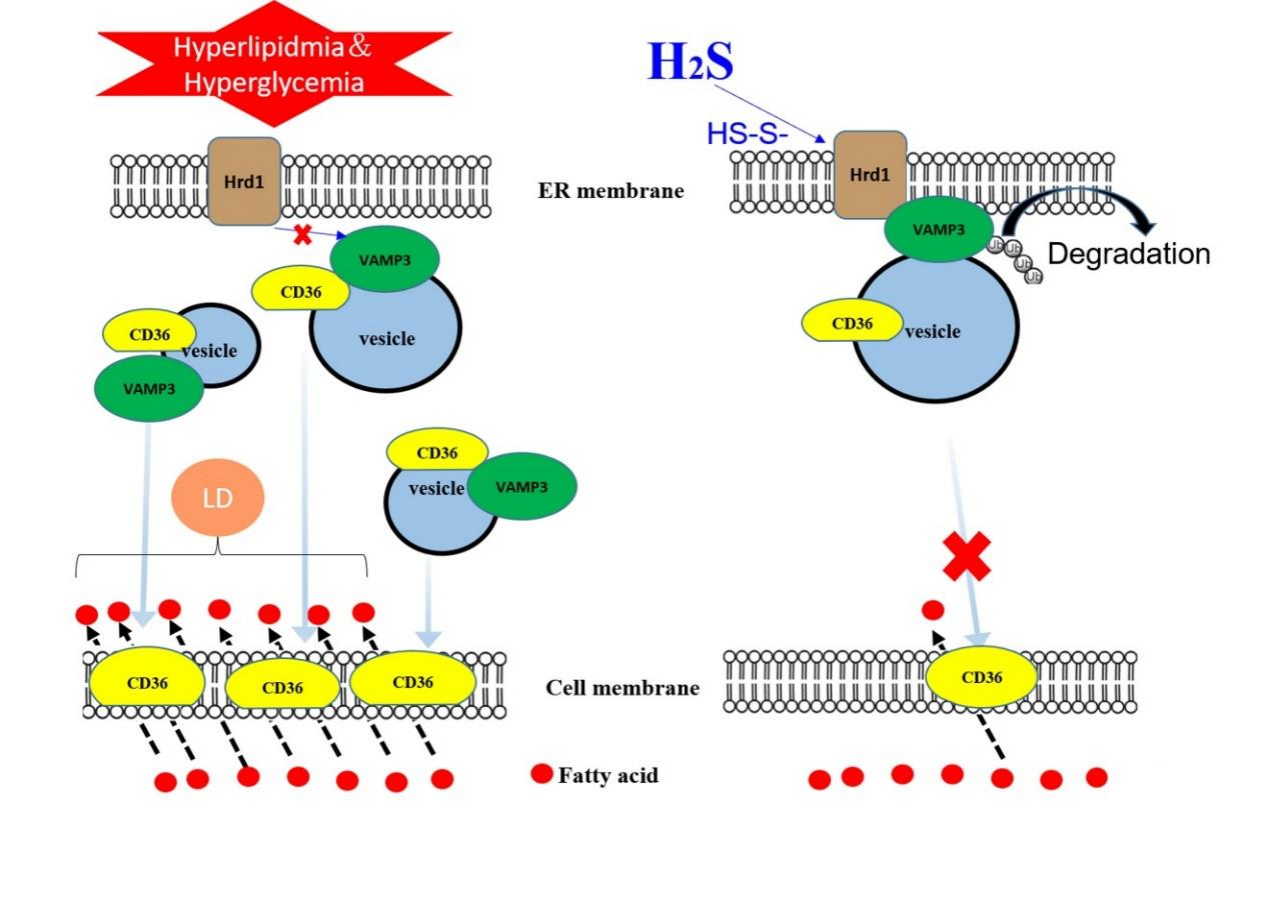
Model for the role of H_2_S in modifying Hrd1 S-sulfhydration to regulate VAMP3 ubiquitylation, which decreases CD36 translocation to the cell membrane. In type 2 diabetic hearts, increased CD36 translocation from the intracellular compartment to the cell membrane is mediated by the SNARE protein VAMP3, which is involved in vesicle formation. Meanwhile, H_2_S mediates S-sulfhydration on Hrd1 (an E3 ubiquitin ligase) at the Cys115 site and then promotes VAMP3 protein degradation by the ubiquitination pathway, thereby inhibiting CD36 translocation to the cell membrane. This inhibition reduces free fatty acid uptake by diabetic cardiomyocytes, thus inhibiting triacylglycerol accumulation in diabetic hearts. H_2_S thus has a certain protective effect on cardiomyocytes in the context of diabetic cardiomyopathy.

To further investigate whether Hrd1 regulates the formation of lipid droplets, we successfully knocked down Hrd1 expression using siRNA in H9c2 cells (Supplementary Fig. 3). Deletion of Hrd1 increased the expression of CD36 in the cell membrane (Fig. 7F) and increased the sizes and numbers of lipid droplets in cells treated with NaHS (Fig. 7G). All results supported the above hypothesis that Hrd1 is involved in regulating fatty acid uptake and lipid droplet formation (Fig. 8).

## DISCUSSION

H_2_S has been suggested to be beneficial for a range of cardiovascular diseases, such as cardiac ischemia and atherosclerosis. Our data revealed that H_2_S levels were reduced in the hearts of db/db mice. A large number of lipid droplets were produced in the db/db mice; however, the number of droplets was reduced by treatment with NaHS, and we thus postulated that H_2_S regulated endocytosis by S-sulfhydration of target proteins.

In the present study, we found that cardiac H_2_S levels and CSE expression were clearly decreased in db/db mice compared to control mice. The number and size of the droplets increased in a time-dependent manner; however, administration of exogenous H_2_S could reduce the numbers and sizes of the droplets in the hearts of db/db mice. These results demonstrated that impairment of the H_2_S/CSE system is involved in dysregulated free fatty acid transportation. We also report a novel finding that H_2_S regulated the interaction between Hrd1 and VAMP3 through Hrd1 S-sulfhydration. Collectively, these data demonstrate that H_2_S plays an important role in modulating VAMP3 ubiquitylation to regulate free fatty acid transporters in the context of diabetes.

The transport of LCFA is primarily a protein-mediated event involving several transport proteins, including CD36 and FABP [1]. Both of these transport proteins have been shown to translocate from an intracellular depot to the plasma membrane in response to stimuli [25, 26]. Accumulating evidence has revealed that SNAREs constitute the mechanistic core complexes of membrane fusion, which regulates intracellular CD36 distribution [27]. Our results showed that the expression levels of CD36 and VAMP3 in cardiomyocyte membranes were significantly increased in db/db mice compared to control mice. A large number of SNARE mammalian proteins have been characterized, such as VAMP3, which has been implicated in the recycling of membrane receptors from endosomes to the plasma membrane. The importance of ubiquitylation in controlling receptor endocytosis and endosomal sorting has been reported in many studies [10, 11]. Ubiquitylation is thought to be a significant signal in the regulation of intracellular trafficking and offers many advantages over other types of signals [12]. Hrd1, a multi-transmembrane RING domain E3 ligase, plays a role in ER protein quality control. Hrd1 is also responsible for the ubiquitylation and degradation of ER and Golgi membranes [28]. Graham and colleagues recently demonstrated that in budding yeast, Tul1, which shares sequence similarity with the Hrd1 E3 ligase complex, participates in the ubiquitylation and recycling of an exocytic SNARE at the early endosome [29]. Our data showed that the expression of Hrd1 was lower in model db/db mice than in db/db mice treated with exogenous H_2_S, and coimmunoprecipitation revealed that Hrd1 interacted with VAMP3 in cardiomyocytes.

Sulfhydration has been proposed as a mode of H_2_S action in which H_2_S adds sulfur atoms to cysteine residues of target proteins to induce conformational changes, leading to modification of target protein activity [30]. According to the amino acid sequences available in PubMed, there are nine cysteines in the Hrd1 sequence. Our results demonstrated that Hrd1 in db/db mice could be modified by sulfhydration upon treatment with exogenous H_2_S. We also found that VAMP3 expression in the cell membrane was clearly reduced in the db/db mice treated with exogenous H_2_S. To further investigate whether exogenous H_2_S affected Hrd1 activity, we overexpressed Hrd1 in which Cys115 was mutated to Ala and demonstrated that treatment with exogenous H_2_S decreased VAMP3 ubiquitylation levels and CD36 translocation to the cell membrane. This finding suggested that sulfhydration of Hrd1 at Cys115 might be one of the detailed mechanisms by which H_2_S reduces CD36 translocation to the cell membrane, reduces LCFA uptake and inhibits lipid droplet formation (Fig. 8).

In conclusion, this study led to the novel finding that H_2_S increases Hrd1 sulfhydration and participates in regulating VAMP3 ubiquitylation to prevent LCFA uptake. Our findings raise the possibility that H_2_S supplementation might be a clinically useful therapeutic strategy to attenuate diabetic cardiomyopathy.

## Supplementary Materials

The Supplemenantry data can be found online at: www.aginganddisease.org/EN/10.14336/AD.2019.0530.
