# Supplementary material for: Exogenous H2S Induces Hrd1 S-sulfhydration and Prevents CD36 Translocation via VAMP3 Ubiquitylation in Diabetic Hearts
Source: Aging Dis. 2020 Mar 9;11(2):286–300. doi: 10.14336/AD.2019.0530 (PMC7069459; doi:10.14336/AD.2019.0530)
Supplement: Supplementary file 1 — The Supplemenantry data can be found online at: www.aginganddisease.org/EN/10.14336/AD.2019.0530. [file AD-11-2-286-s.pdf]

SUPPLEMENTARY DATA

Exogenous H_2S Induces Hrd1 S-sulfhydration and Prevents CD36 Translocation via VAMP3 Ubiquitylation in Diabetic Hearts

Miao Yu[1], Haining Du[1], Bingzhu Wang[1], Jian Chen[1], Fangping Lu[1], Shuo Peng[1], Yu Sun[1], Ning Liu[1], Xiaojiao Sun[1], Shiyun Dong[1], Yajun Zhao[1], Yan Wang[2], Dechao Zhao[3], Fanghao Lu[1, *], Weihua Zhang[1,4, *]

[1]Department of Pathophysiology, Harbin Medical University, Harbin, China.
[2]Department of Urologic Surgery, First Affiliated Hospital of Harbin Medical University, Harbin, China.
[3]Department of Cardiology, First Affiliated Hospital of Harbin Medical University, Harbin, China.
[4]Key Laboratory of Cardiovascular Medicine Research (Harbin Medical University), Ministry of Education, Harbin, China.

© 2019. Yu M et al. Published online at http://www.aginganddisease.org/EN/10.14336/AD.2019.0530

SUPPLEMENTARY DATA

METHODS

Measurement of 2-NBDG uptake

Glucose uptake was determined by a nonradioactive method using a new fluorescent analog of 2-deoxy-2-[(7-nitro-2,1,3-benzoxadiazol-4-yl) amino]-D-glucose (2-NBDG) (Sigma). Cells were seeded and treated for 48 hours in 96-well plates and washed three times with PBS. Then, the culture medium was removed from each well and replaced with 500 µL of glucose-free DMEM, and the cells were incubated in the presence of 10 µmol/L 2-NBDG at the indicated concentrations for 40 min. The fluorescence intensity was recorded at excitation and emission wavelengths of 485 nm and 535 nm, respectively.

Measurement of fatty acid uptake

Briefly, cells were grown on coverslips in 12-well plates, and after treatment, the cells were serum-starved for 5 hours. The medium was aspirated, and the cells were washed twice with PBS containing fatty acid-free albumin. The cells were then incubated with BODIPY 558/568C12 (1 µmol/L) for 2 min at 37 °C, and the coverslips were washed 3 times with PBS and mounted on clean glass slides using Dako antifade solution (Dako Corp, Carpinteria, CA). For confocal microscopy analysis, BODIPY-conjugated fatty acids were excited at 488 nm with a fluorescence microscope (Olympus XSZ-D2, Japan). Fluorescent images were obtained using FluoView software, and the fluorescence intensity was quantitated with ImageJ software.

Glucose tolerance test analysis

Mice were intraperitoneally injected with D-glucose (2 g/kg mass). Tail blood was collected, and blood glucose was determined using a glucometer.

Echocardiography analysis

Mouse cardiac function was assessed using an echocardiography system (GE VIVID 7 10S, St. CT., Fairfield, USA) after 6 weeks, 12 weeks and 20 weeks of treatment with NaHS. The mice were lightly anesthetized with Avertin at a dose of 240 mg·kg-1, and the mouse body temperature was maintained as close to 37 °C as possible during the entire process. Left ventricular parameters were measured, including EF % and FS %.

HPLC-MS/MS analysis

Based on our previous study, we identified lysine-ubiquitylated proteins in cardiac tissues of db/db mice and in cells treated or not treated with NaHS [1]. The peptides were dissolved in 0.1% FA and directly loaded onto a reversed-phase precolumn (Acclaim PepMap 100, Thermo Scientific). Peptide separation was performed using a reversed-phase analytical column (Acclaim PepMap RSLC, Thermo Scientific). The gradient was composed of an increase in solvent B (0.1% FA in 98% ACN) from 6% to 22% for 22 min, an increase from 22% to 36% for 8 min, an increase to 80% over 5 min, and a hold at 80% for the last 3 min. All steps were conducted at a constant flow rate of 300 nL/min on an EASY-nLC 1000 UPLC system. The resulting peptides were analyzed with a Q Exactive^{TM} Plus Hybrid Quadrupole-Orbitrap Mass Spectrometer (Thermo Fisher Scientific).
The peptides were subjected to an NSI source followed by MS/MS in a Q Exactive^{TM} Plus (Thermo) coupled online to UPLC. Intact peptides were detected in the Orbitrap at a resolution of 70,000. Peptides were selected for MS/MS using a normalized collision energy (NCE) setting of 28, and ion fragments were detected in the Orbitrap at a resolution of 17,500. A data-dependent procedure that alternated between one MS scan and 20 MS/MS scans was applied for the top 20 precursor ions above a threshold ion count of 2E4 in the MS survey scan with 10.0 s dynamic exclusion. The electrospray voltage applied was 2.0 kV. Automatic gain control (AGC) was used to prevent overfilling of the ion trap; 5E4 ions were accumulated for generation of MS/MS spectra. For MS scans, the m/z scan range was 350 to 1800.

Protein sequence alignments and bioinformatics analysis

© 2019. Yu M et al. Published online at http://www.aginganddisease.org/EN/10.14336/AD.2019.0530

SUPPLEMENTARY DATA

To characterize the identified lysine-ubiquitylated proteins, a series of bioinformatics tools were used for protein annotation and functional analysis. Specifically, Gene Ontology (GO) functional annotation, Kyoto Encyclopedia of Genes and Genomes (KEGG) pathway analysis, domain annotation and subcellular localization prediction were performed. GO annotation was based on three categories: biological process, cellular component and molecular function. The KEGG online service tools of the KEGG map were used to annotate the proteins with KEGG pathway descriptions. Domain annotation was performed using InterProScan on the InterPro domain database via Web-based interfaces and services. For each category of proteins, the InterPro database (a resource that provides functional analysis of protein sequences by classifying them into families and predicting the presence of domains and important sites) was searched using the functional annotation tool of the Database for Annotation, Visualization and Integrated Discovery (DAVID) against the background of *Homo sapiens*. A two-tailed Fisher's exact test was employed to test the enrichment of the protein-containing IPI entries against all IPI proteins. Correction for multiple hypothesis testing was conducted using standard false discovery rate control methods, and domains with a corrected p-value < 0.05 were considered significant.

References

1. Sun X, Zhao D, Lu F, Peng S, Yu M, Liu N, Yu Sun1, et al. (2019). Hydrogen Sulfide Regulates Muscle RING Finger-1 Protein S-Sulfhydration at Cys44 to Prevent Cardiac Structural Damage in Diabetic Cardiomyopathy. Br J Pharmacol, doi: 10.1111/bph.14601.

© 2019. Yu M et al. Published online at http://www.aginganddisease.org/EN/10.14336/AD.2019.0530

SUPPLEMENTARY DATA

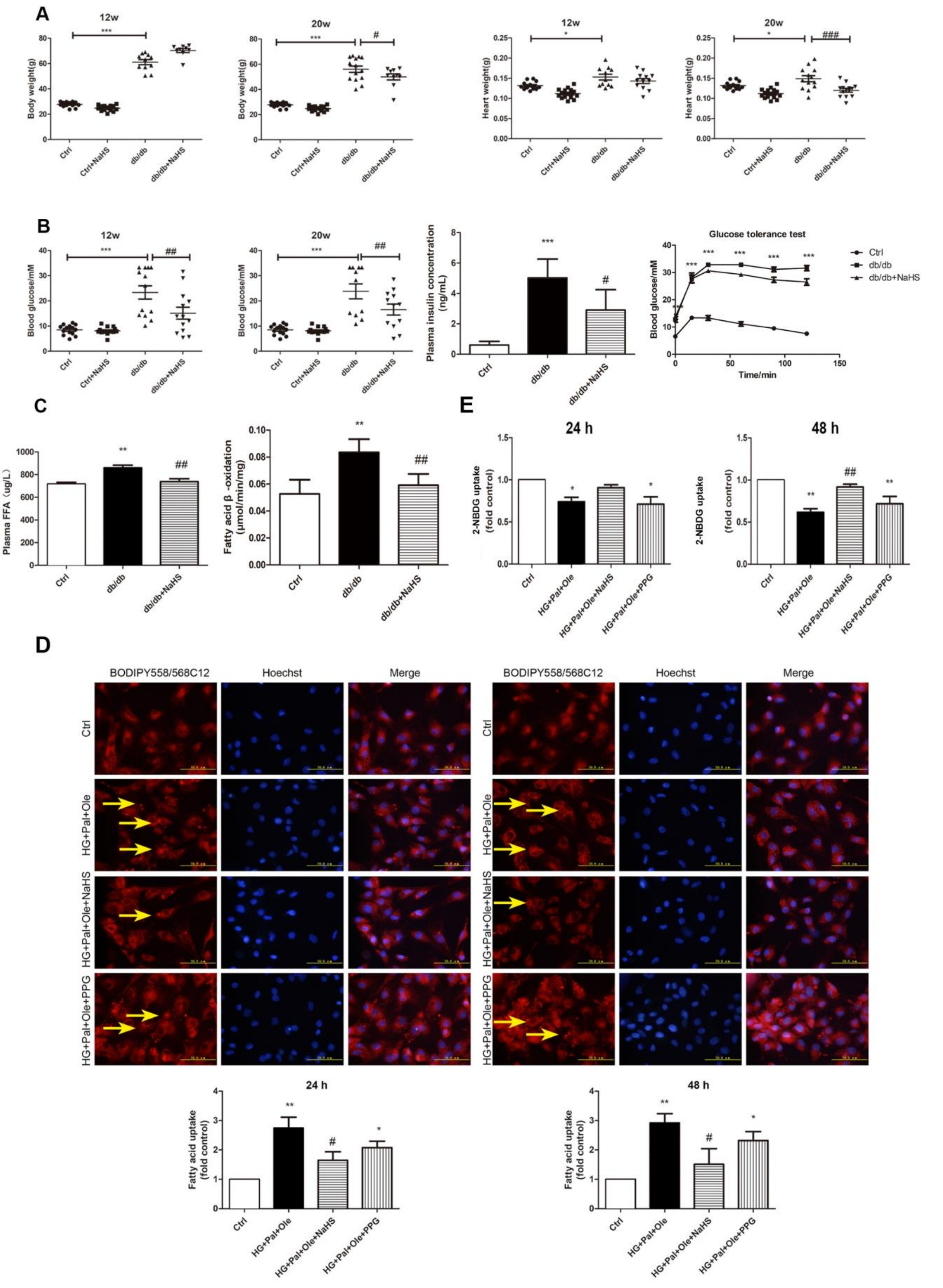

© 2019. Yu M et al. Published online at http://www.aginganddisease.org/EN/10.14336/AD.2019.0530

SUPPLEMENTARY DATA

Supplementary Figure 1. General indexes of the db/db mice. (A) The body and heart weights of 12- to 20-week–treated control mice, control+NaHS mice, db/db mice and db/db+NaHS mice. (B) The blood glucose concentrations of 12- to 20-week–treated mice and the plasma insulin concentrations of 20-week–treated mice were measured. Intraperitoneal glucose tolerance tests were performed on three groups of mice injected with 2 g glucose/kg. (C) The free fatty acid plasma levels and β-oxidation in 20-week–treated db/db, control and db/db+NaHS mice. (D) Fatty acid uptake (yellow arrow) was measured with the BODIPY 558/568C12 probe in NRCMs at 24 and 48 hours. (E) 2-NBDG uptake in NRCMs at 24 and 48 hours. Data are presented as the mean± SD from n=7 replicates. *p<0.05, **p<0.01, ***p<0.001 vs control, #p<0.05, ##p<0.01, ###p<0.001 vs db/db or HG+Pal+Ole group.

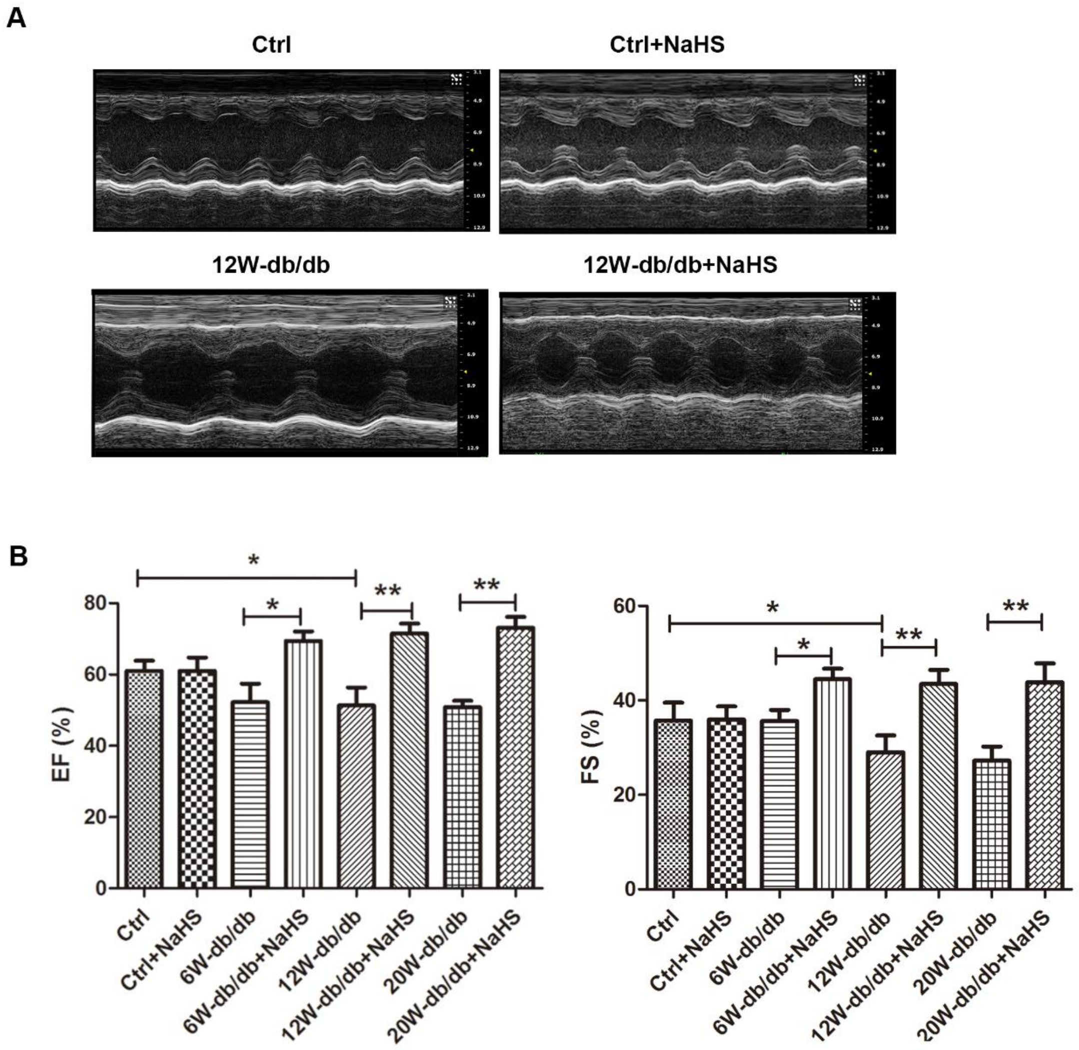

Supplementary Figure 2. Exogenous H_2S protected cardiac function in db/db mice. Representative M-mode echocardiograms of mouse hearts and quantitative analysis of EF and FS by echocardiography after 6 weeks, 12 weeks and 20 weeks of NaHS treatment. Data are presented as the mean± SD from n=4 replicates. *p<0.05, **p<0.01.

© 2019. Yu M et al. Published online at http://www.aginganddisease.org/EN/10.14336/AD.2019.0530

SUPPLEMENTARY DATA

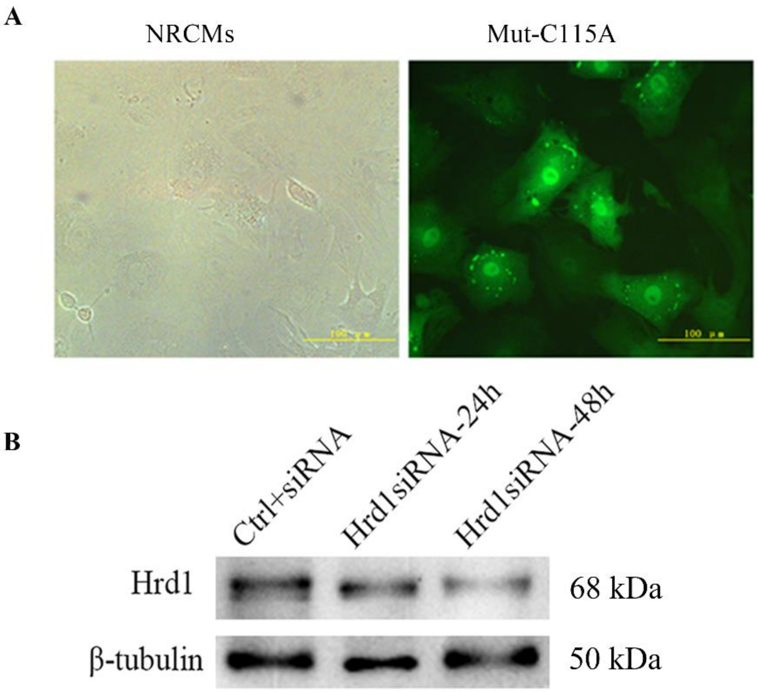

Supplementary Figure 3. Successful mutation and interference. (A) Hrd1 mutated at Cys115 was successfully transfected into neonatal rat cardiomyocytes for 48 hours. The NRCMs are shown in the left picture (Olympus XSZ-D2), and in the right picture, the GFP (green fluorescence) in the same location is shown, as detected using a fluorescence microscope (Olympus, XSZ-D2). (B) Hrd1 was knocked down with siRNA in H9c2 cells, and the results of Western blot analysis confirm that the expression of Hrd1 was downregulated in the knockdown group compared with the control group.

© 2019. Yu M et al. Published online at http://www.aginganddisease.org/EN/10.14336/AD.2019.0530
